# Therapeutic Potential of Natural Killer Cells in Gastric Cancer

**DOI:** 10.3389/fimmu.2018.03095

**Published:** 2019-01-21

**Authors:** Yu Du, Yongchang Wei

**Affiliations:** Department of Radiation and Medical Oncology, Zhongnan Hospital, Wuhan University, Wuhan, China

**Keywords:** natural killer cells, gastric cancer, immunity, adoptive therapy, checkpoint blockades

## Abstract

Gastric cancer (GC) is one of the most common cancers, with a high incidence of cancer death. Despite various therapeutic approaches, the cures and prognosis of advanced GC remain poor. Natural killer (NK) cells, which are known as important lymphocytes in innate immunity, play vital roles in suppressing GC initiation, progression, and metastases. A wide range of clinical settings shows that increasing the number of NK cells or improving NK cell antitumor activity is promising in GC patients. NK cell adoptive therapy (especially expanded NK cells) is a safe and well-tolerated method, which can enhance NK cell cytotoxicity against GC. Meanwhile, cytokines, immunomodulatory drugs, immune checkpoint blockades, antibodies, vaccines, and gene therapy have been found to directly or indirectly activate NK cells to improve their killing activity toward GC. In this review, we summarize recent advancements in the relationship between NK cells and GC and point out all the innovative strategies that can enhance NK cells' function to inhibit the growth of GC.

## Introduction

Gastric cancer (GC) was the world's third-leading cause of cancer death in 2012, resulting in 723,000 deaths (1). GC rates in men are nearly twice as high as those in women, and they vary widely across countries, with the highest incidence rates in Eastern Asia (particularly in China), Central and Eastern Europe, and Central and South America (1, 2). Although the incidence and mortality of GC have declined in recent years due to standardized surgical techniques, innovations in clinical diagnosis, and the development of new chemotherapy regimens, the survival rate for advanced GC remains low across the world. Therefore, investigating the molecular biology of GC to realize novel effective therapeutic approaches results in beneficial progress in the treatments of GC.

Natural killer (NK) cells are vital members of innate immunity and can recognize and kill tumors and infected cells as well as produce many cytokines to regulate adaptive immunity (3). Meanwhile, similar to cytotoxic T lymphocytes, NK cells also preserve specific memories after encountering a pathogen. Experienced NK cells show a robust protective response to reactivation by the initial pathogen but also by other pathogens (4, 5). However, the amount, subsets, cytokines, and cytotoxicity of NK cells are decreased in GC patients and impair the immune system severely (6). Immunotherapy, which has made some breakthroughs in many cancers, has been introduced as a modality to improve the function of the immune system in GC. Among immunotherapies, therapies targeting the activation of NK cells and enhancement of NK cell activity are under research, shedding light on the treatments of GC. Recent evidence shows that the signal transducer and activator of the transcription family 5 target CIS, interleukin (IL)-1R8, transforming growth factor (TGF)-β, and adenosine all function as inhibitors of NK cells. Better understanding of these suppressive pathways is helpful for discovering effective candidates for the therapeutic manipulation of NK cells (7). Here, we discuss the relationship between GC and NK cells and review the research progress of NK cell immunotherapy for GC.

## Characteristics OF NK Cells

NK cells play important roles in host innate immunity with high antitumor, antiviral, and antimicrobial activity and contribute to the activation and regulation of adaptive immune responses (8, 9). About 3–5% of human peripheral blood lymphocytes are NK cells. These cells react much faster than T cells upon stimulation, as they do not need previous sensitization, antibody binding, or pathogen presentation.

Phenotypically, NK cells are defined by the expression of CD56 and the lack of CD3. CD56^dim^ CD16^bright^ NK cells, which account for 90% of NK cells, are mature, which means they mediate antibody-dependent cellular cytotoxicity (ADCC), exhibiting high levels of perforin and enhanced killing. The remaining 10% of NK cells producing various cytokines are immature, which express CD56^bright^ CD16^dim^ or CD56^bright^ CD16^−^ (3, 10). The antitumor activity of NK cells is mostly determined by a set of inhibitory and activating receptors (11). Inhibitory receptors include killer-cell immunoglobulin-like receptors (KIRs) that bind to class-I human leukocyte antigen (HLA) molecules or inhibitory C-type lectins Ly49s and the heterodimer CD94/NKG2A-B, which recognize HLA-E molecules. Some activating receptors belong to the Ig-like family, such as CD16, NKp46, NKp30, and NKp44. Moreover, the C-type lectin receptors, including CD94/NKG2C-E (recognizing HLA-E) and NKG2D (recognizing non-classical HLA), also activate receptors (12–14). By interacting with target cells, NK cell activity is changed.

**Figure 1:**
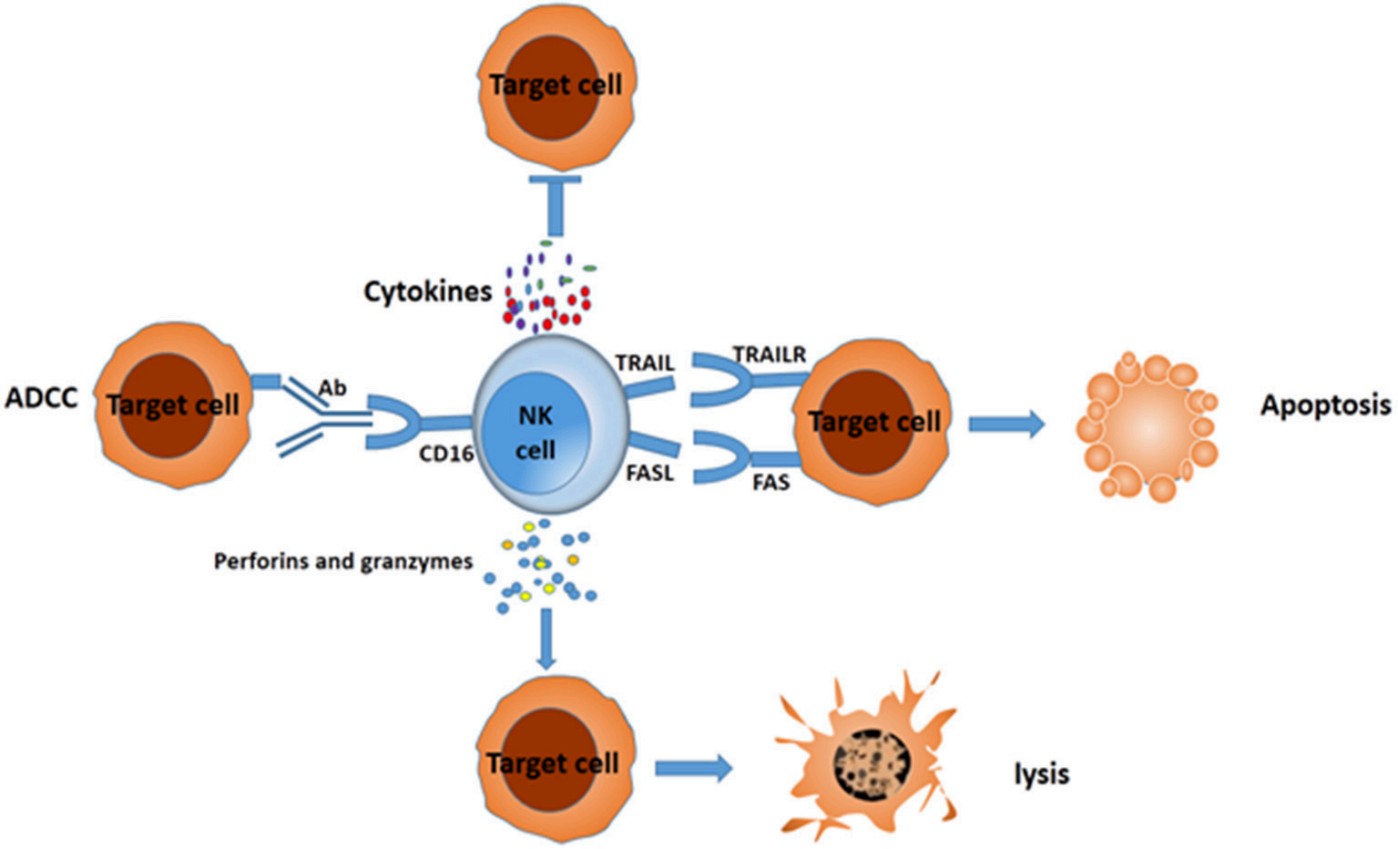
Different mechanisms for NK cells to suppress target cells. NK cells can mediate the death of target cancer cells by ADCC, secreting IFN-γ and TNF-a, releasing perforin and granzymes, or eliciting apoptosis via formation of complex FAS/FASL and TRAIL/TRAILR.

Emerging evidence shows that cancers develop multiple strategies to escape CD8^+^ T cell recognition, but they can be preferentially attacked by NK cells (15). NK cells eliminate target cells through several different mechanisms (Figure 1). After adhering to the target cells, NK cells release many cytotoxic granules containing perforin and granzymes, which lead to cell lysis. NK cells express tumor necrosis factor (TNF)–related apoptosis-inducing ligand family (TRAIL) and Fas-Ligand (FASL) (CD95L), which interact with TRAIL receptors and FAS in target cells, respectively. The interaction leads to the formation of a death-inducing signaling complex and elicits apoptosis (10). NK cells can also secrete sufficient amounts of the cytokines interferon (IFN)-γ (16) and TNF-α (17) to increase cytotoxicity. In addition, one potent activating receptor, CD16 (FcγIIIA), can recognize Fc on human IgG1 antibodies and trigger ADCC (18). All these cytotoxicity mechanisms enable NK cells to eliminate different types of tumor cells.

## NK Cells and GC

**Figure 2:**
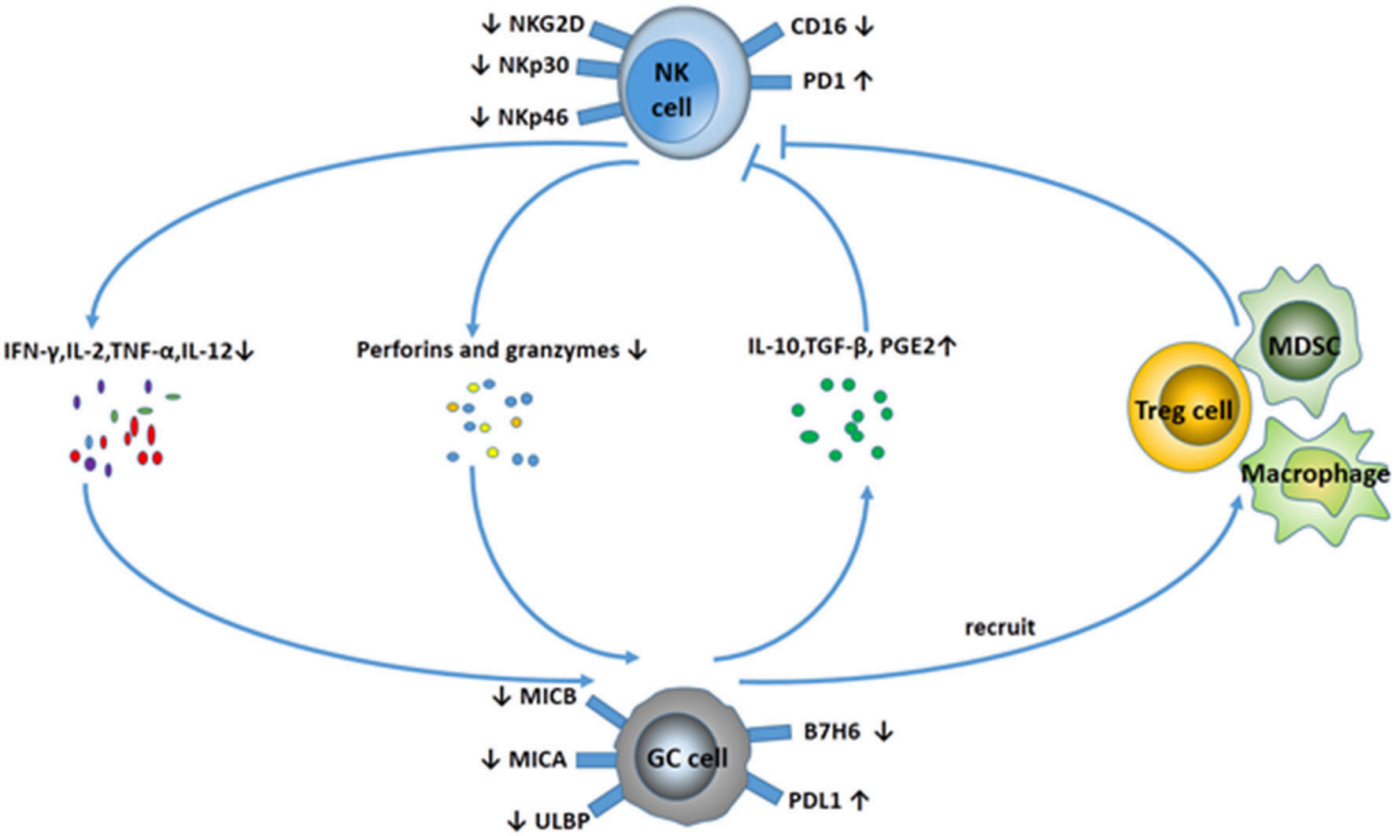
Interaction between NK cells and GC. NK cells display a suppressive phenotype with fewer activating receptors (NKG2D, NKp30, NKp46) and higher expression of PD-1 in GC patients. In addition, NK cells secret fewer cytokines (IFN-γ, IL-2, TNF-α, IL-12), and the ability to release perforins and granzymes is decreased. Meanwhile, GC cells downregulate the expression of MICA/B, ULBP, and B7H6 to avoid NK cell-mediated innate immunity. GC cells can also release some cytokines, including IL-10, TGF-β, and PGE2, and recruit MDSCs and Treg cells to suppress NK cell activity.

Compared with tumor-specific cytolytic T cells, NK cells can kill tumors with low or absent HLA class I expression. Notably, NK cells could effectively be activated by cancer stem cells (CSCs). CSCs are responsible for tumor relapses, as they are resistant to chemo- and radiotherapy, because of their quiescent status. However, a study found that gastric CSCs can be killed by NK cells via CD133 in an NKG2D-dependent manner (19). An increasing amount of data show there is an important relationship between NK cells and the progression of GC. With the progression of GC, the number and the function of NK cells decrease sharply, which leads to the malignancy of GC in reverse. The interaction between NK cells and GC can be visualized in Figure 2.

### NK Cell Dysfunction in GC Patients

A study demonstrated that the frequency of apoptotic NK cells in GC patients (21.3 ± 11.6%) was increased significantly compared with normal controls (11.2 ± 5.2%; *p* = 0.0016), and their frequencies were related to the progression of GC (20). NK cell infiltration in intratumoral regions is significantly decreased, which is associated with decreased survival and disease progression in GC patients (21, 22). Gulubova et al. elucidated that the number of NK cells was decreased in patients with gastric and colorectal cancer with liver metastases compared with those without liver metastases (10.1 ± 11.6% vs. 16.6 ± 8.9%, *p* = 0.039) (23). The percentages of NK cells in blood as well as NK cell activity were significantly increased after gastrectomy (24).

NK cell activity is damaged in GC patients. Data show that there is an evident association between NK cell activity and some clinicopathological parameters, including tumor volume, clinical stage, lymphatic and vascular invasion, and lymph node metastases in GC (25, 26). In GC patients, NK cells show a suppressive phenotype, with downregulated expression of activating receptors and upregulated expression of inhibitory receptors. In particular, NKG2D is a key receptor for NK cell activation and has multiple ligands, including MHC class I chain-related A (MICA), MICB, and several UL-16–binding proteins (27). Yoshimura et al. investigated 98 GC patients who underwent surgery from 2004 to 2008. They found that patients with NKG2D expression in tumors had significantly longer overall survival (OS) than patients without NKG2D expression in tumors (*p* = 0.0217), and the longest OS was observed in patients positive for ULBP1 and NKG2D (28, 29). Except for downregulated receptors of NKG2D, NKp30, and NKp46, NK cells also release fewer cytotoxic granules of perforin and granzyme B and are characterized by decreased IFN-γ, TNF-α, and Ki-67 expression in GC patients (22, 30). In addition, TNF-α, IL-2, T-bet, and IL-15Rβ levels were decreased in NK cells from the GC tissue and peripheral blood in the GC patients, leading to a decrease in the function of NK (6). Moreover, Kono et al. discovered that NK cell dysfunction contributed to the impaired Herceptin-mediated ADCC in advanced GC patients, which was correlated with the downregulation of CD16zeta expression (31).

### Strategies for GC to Escape From NK Cell-Mediated Immunity

GC develops various measures to escape from innate immune response based on NK cells. NK cells play their roles mainly by the interaction between immunoregulating receptors and the ligands. Some GC cells express fewer NKG2D ligands to decrease NK cell sensitivity. The NKG2D ligand expression in GC patients is associated with favorable presenting features and a better OS (32). Patients with GC release higher levels of soluble MICA and MICB compared with healthy donors to downregulate NKG2D expression and dampen NK cell cytotoxicity (33). In addition, Xing et al. demonstrated that the sensitivity of GC cells to the cytotoxicity of NK cells was determined by copy number variations of HLA-I and activation of the NKp30 pathway (34). B7-H6, a human receptor, alerts innate immunity to cellular transformation via its interaction with the NKp30 (35). Chen et al. discovered that B7-H6–positive carcinomas were significantly associated with a higher differentiation, whereas there was no significant difference between B7-H6 expression and prognosis of GC patients (36). In addition, as a non-classical MHC-I antigen, HLA-G is expressed in most of GC tissues. The overexpression of HLA-G in GC cell lines inhibits the cell proliferation and cytotoxic activity of NK-92MI cells and reduces the secretion of IFN-γ and TNF-α through immunoglobulin-like transcript 2 (37).

In addition to ligand expression, GC achieves immunosuppression through suppressive cytokines and cells in its tumor microenvironment. Development of GC is accompanied by augmented levels of serum IL-10 and TGF-β1, which result in a remarkable decrease in cytotoxic activity of NK cells (38). Recently, TGF-β was discovered to convert NK cells into intermediate type 1 innate lymphoid cells (intILC1s) and ILC1s to help tumor escape immunosurveillance (39), whereas the signal transducer SMAD4 impedes the conversion by curtailing non-canonical TGF-β signaling (40). A study suggested that the production of prostaglandin E2 by GC cells may play a primary role in suppressing NK cell proliferation and inducing apoptosis (21). Midkine, a heparin-binding growth factor overexpressed in various human cancers, upregulates MICA/B serum levels of GC patients and inhibits CD107a and granzyme B expression, thereby suppressing NK cell cytotoxicity (33). The neoplastic cells can also evade immune surveillance via generation of regulatory cells, such as Tregs and macrophages, myeloid-derived suppressor cells (MDSCs) (41). A study by Choi et al. showed that an increased proportion of MDSCs was an adverse independent prognostic factor in GC (42). Moreover, the tumor-associated macrophages (TAMs) also play important roles in immune suppression in GC (43). They differentiate into M1 or M2 subtypes according to the stimulus present in the tumor microenvironment (44). M1 TAMs exert antitumor activities by releasing proinflammatory cytokines, whereas M2 TAMs may drive local immune suppression through production of IL-10 and TGF-β. Peng et al. demonstrated that TAMs were physically close to NK cells, and they could impair NK cell expression of IFN-γ, TNF-α, and Ki-67 via producing TGF-β1 (22).

## NK Cell Adoptive Therapy Against GC

Although different treatment strategies have been evaluated in recent years, such as adjuvant chemotherapy, adjuvant chemoradiotherapy, and perioperative chemotherapy, the outcome of GC is still not good. Therefore, investigating innovative therapeutic strategies is of great importance. Adoptive cellular immunotherapy has made some achievements in the treatment of GC (45). NK cells are considered to be promising effector cells in the adoptive immunotherapy of cancer (46) and have been used as an effective treatment modality for hematological malignant diseases and solid tumors (47).

Unlike T-cell infusion, donor-vs.-recipient NK cells reduce leukemia relapse and do not induce graft-vs.-host disease (GVHD) at the same time, as the inhibitory KIR (donor)–HLA-I (patient) mismatch leads to alloreactivity, and then NK cells lyse leukemia blasts, recipient dendritic cells (DCs), and recipient T cells (48–50). Re et al. reported that cancer cells from a GC patient who did not possess at least one of the major HLA class I allele groups were killed efficiently by NK cells (51). Although all published studies agree that NK cell infusion is a safe and well-tolerated procedure and is not associated with GVHD, results of NK cell infusion are not optimal (10, 52). One of the obstacles for NK cell-based treatments is that the exiguous amount of NK cells in blood cannot overcome the large tumor burden; hence, expanding NK cells *in vitro* to obtain a high number of NK cells with high cytotoxicity in compliance with good manufacturing practices (GMP) could be an active measure (10).

**Figure 3:**
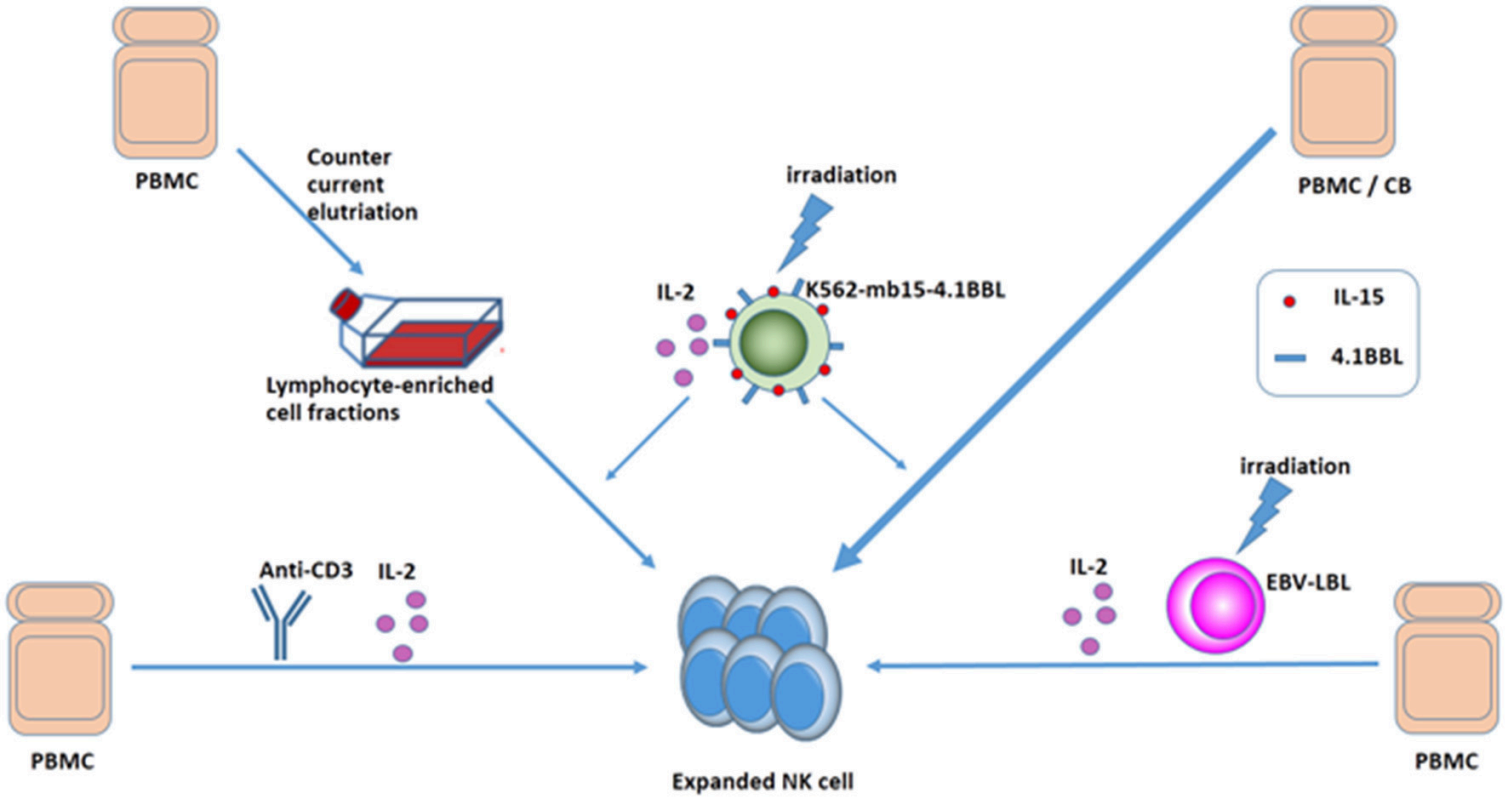
Ways to expand NK cells. Expanded NK cells can be generated from PMBC or CB: (1) culture with cytokines and anti-CD3 without feeder cells, (2) culture with cytokines and irradiated EBV-LCL, and (3) GMP-compliant countercurrent elutriation, and (4) using an APC K562-based system.

Clinical-grade NK cells can be produced from various sources, including peripheral blood, cord blood, bone marrow, and embryonic stem cells (10, 53). Effective amplification of NK cells was achieved by short-term culture with cytokines alone or by co-culture with cytokines and different feeder cells (54–57). In 2008, Alici et al. added anti-CD3 antibody for the first 5 days and IL-2 for the remaining days to peripheral blood mononuclear cells (PBMCs) from healthy individuals and managed to obtain a good quantity of activated NK cells without the need for feeder cells (58). Intriguingly, Sutlu et al. optimized the expansion of clinical-grade NK cells from PBMCs of healthy individuals using an automated bioreactor. The end product of the expansion protocol had a median of 38% NK cells, ensuring that a clinically relevant cell dose was reached (mean 9.8 × 10^9^ NK cells) (59). Moreover, with repeated stimulation of irradiated Epstein-Barr virus-transformed lymphoblastoid cell lines and IL-2 as well as addition of IL-21 at the initiation of the culture, NK cells obtained a 10^11^-fold expansion after 6 weeks. The expanded NK cells upregulate TRAIL, NKG2D, and DNAM-1 and have superior cytotoxicity against tumor cell lines *in vitro* (60). Interestingly, with osteoclasts as feeder cells, highly potent NK cells can be obtained in a considerable frequency (61, 62). Several studies suggested that using an artificial antigen-presenting cell K562-based system to expand NK cells from core blood units or PBMCs was another efficient and safe technique, which enabled us to get plenty of NK cells off the shelf (56, 63–66). While clinical studies are ongoing using elutriation-derived monocytes for large-scale generation of DCs to treat a variety of metastatic cancers, Voskens et al. demonstrated that cytolytic NK cells could be generated from lymphocyte-enriched fractions obtained by GMP-compliant countercurrent elutriation from PBMCs (56). NK cells can also be generated from hematopoietic stem cells (67). In addition to donor-derived primary NK cells, cytotoxic cell lines, such as NK-92 have also been developed for clinical applications. Continuously expanding NK-92 cells do not require laborious isolation from blood, and sufficient NK cells with unlimited availability can be obtained (68). Sakamoto et al. successfully generated large numbers of activated NK cells by stimulating PBMCs of patients with digestive cancer, including GC with OK432, IL-2, and modified FN-CH296-induced T cells. The expanded cells were safe to administer in a monotherapy (69). Different methods of obtaining a large quantity of NK cells are shown in Figure 3.

In most instances, the expanded NK cells alter the balance of receptor expression and cytotoxicity, restoring cytotoxicity against both various allogeneic tumor targets and, more importantly, against autologous-derived gastric tumor targets. After expansion, NK cells significantly upregulate activating receptors DNAM-1, NKp46, NKp44, NKp30, and NKG2D and express high levels of CD16 as well as TRAIL and FasL. In addition, they rapidly release large amounts of IFN-γ and TNF-α after stimulation and efficiently kill tumor cells (13, 54, 56). Mimura et al. found that resting NK cells from GC patients showed negligible cytotoxicities against all GC cell lines. IL-2–stimulated NK cells showed variable cytotoxicities, which remained below 30% for most cell lines tested, and NK cells expanded by coculture with K562-mb15-4.1BBL cells were markedly cytotoxic, with the mean cytotoxicity exceeding 65% in three of the eight GC cell lines tested (32). These findings suggest that expanded NK cell-adoptive therapy could be used to augment the antitumor effects of endogenous NK cells.

## Therapies to Improve NK Cell Function

**Figure 4:**
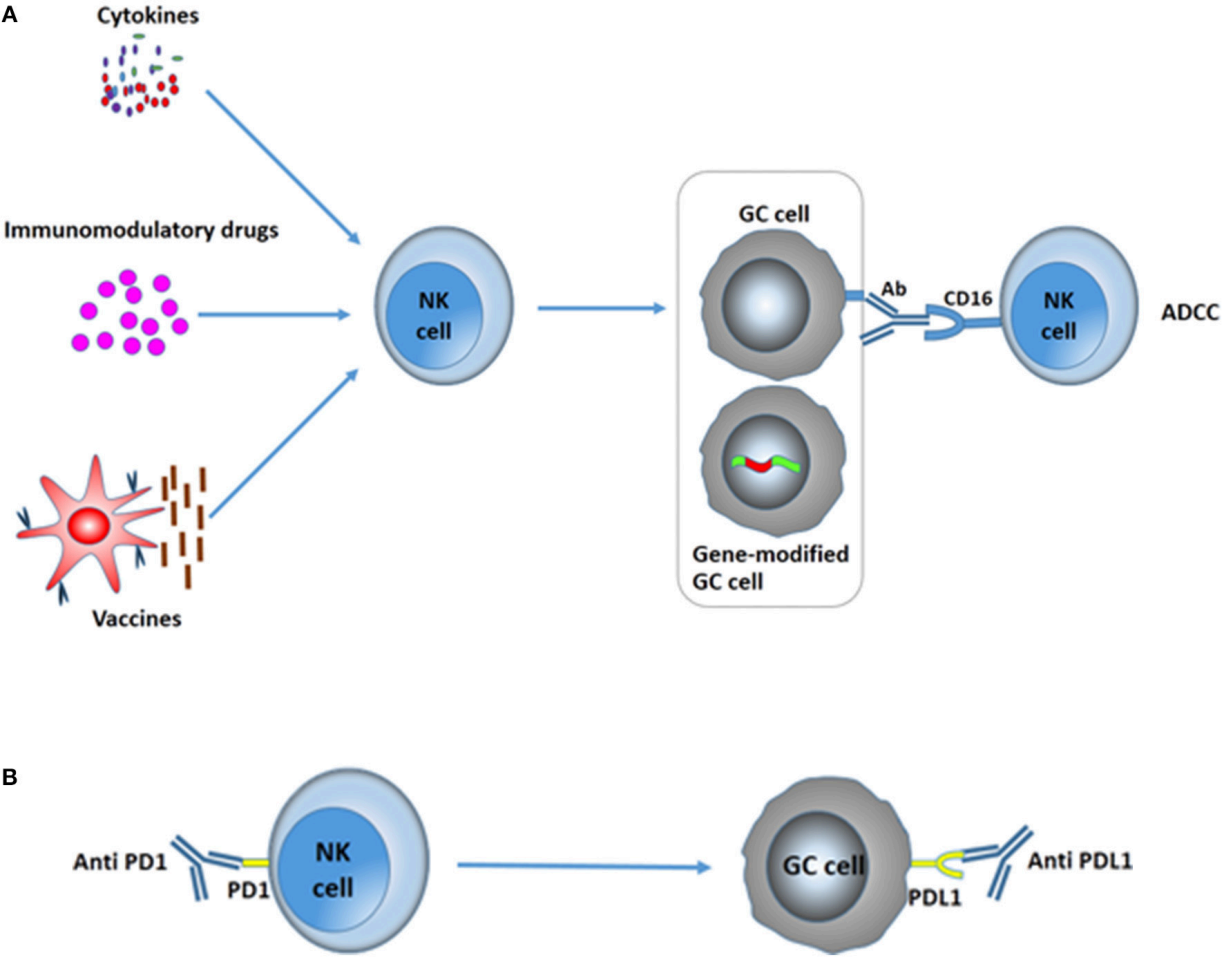
Various immunotherapies in the treatment of GC to improve NK activity. **(A)** Cytokines, immunomodulatory drugs, and vaccines can increase the cytotoxicity of NK cells to kill GC cells. Moreover, antibodies can markedly increase NK cell-mediated ADCC, resulting in the death of GC cells. Gene therapy toward GC cells could improve the immunogenicity or susceptibility of gastric tumor cells to NK cells. **(B)** Immune checkpoint blockades. PD-1/PD-L1 blockades significantly augment degranulation and IFN-γ secretion of NK cells and suppress the evasion of GC.

So far, different immunotherapy approaches including vaccines, monoclonal antibodies, cytokines, and cellular adoptive therapy have been proven to directly stimulate and activate immunity and raise the number of effective cells or cytokines to strengthen the immune response or increase the immunogenicity or susceptibility of cancer cells. Besides NK cell-adoptive therapy, other immunotherapies that can manipulate NK cells and enhance NK cell activity to improve immune responses hold great promise for GC (Figures 4A,B).

### Cytokines Reverse NK Cell Dysfunction in GC

With the progression of tumors, NK cells often become anergic due to downregulation of activating receptor signaling and upregulation of inhibitory receptors as well as suppressive regulatory cells or soluble factors in the microenvironment. Therefore, avoiding NK cell exhaustion would be an effective modality to cure cancer. Treatment with activating cytokines or blocking the signaling of suppressive cytokines might reverse NK cell exhaustion in tumors and chronic infections (14).

Treatments with NK-activating cytokines, IL-12, IL-18, or a mutant form of IL-2 (the “superkine” called H9), restored effector functions of MHC-I-deficient tumor-infiltrating NK cells with impaired signaling downstream of activating receptors. Finally, the survival of MHC-I-deficient tumor-bearing mice was increased (70). In addition, a human gastric carcinoma cell line HR, transduced with the IL-2 gene, could secrete sufficient quantities of bioactive IL-2. Thus, it became more susceptible than parental tumor cells to NK cells, and hepatic metastases in tumor-bearing mice regressed due to the recruitment of NK cells to the tumor site (71). Garcia-Lora et al. found that compared with unstimulated NKL cells, IL-2-stimulated NK cells obtained sustained growth and cytolytic activity by regulating different nuclear transcription factors, protein kinase C isoenzymes and mitogen-activated protein kinases (72, 73). In addition, IL-2 *ex vivo* treatment of NK cells could restore the impairment of Herceptin-mediated ADCC in patients with GC, concomitant to the normalization of the expression of CD16zeta molecules (31). Another cytokine, IL-15, can promote the survival and expansion of NK cells mainly through various STAT5 species. NK cells expressing membrane-bound IL-15 achieved autonomous growth and increased cytotoxicity (7, 74). Moreover, IL-15 fused with the extracellular domain of NKG2D (dsNKG2D–IL-15) exhibited enhanced NK cell tumor infiltration and higher efficiency than IL-15 in suppressing xenografted GC growth in nude mice (75). On the other hand, fractalkine (CX3CL1), a CX3C chemokine, could enhance the recruitment of NK cells and induce both innate and adaptive immunity, thereby yielding a better prognosis in gastric adenocarcinoma (76). Cytokines that reverse NK cell dysfunction are illustrated in Table 1.

**Table 1:** Cytokines reverse NK cell anergy in GC.

**Cytokine**	**Function**	**Molecular mechanisms**	**Trials**	**References**
IL-2	Improve NK cell–mediated ADCC Increase IL-2 gene–transduced HR susceptibility Recruit NK cells	Restore CD16zeta expression of NK cells Lower Lower expression of HLA-I molecules on tumor cells	Patients, cell lines Tumor-bearing mice, cell lines	(31) (71)
IL-5	Activate NK cells Promote proliferation and cytotoxicity of NK cells	Increase CD69 and CD107a expression Elevate IFN-γ production production	Tumor-bearing mice, cell lines	(75)
dsNKG2D–IL-15	Activate NK cells Promote proliferation and cytotoxicity of NK cells	Efficiently bind to MICA Increase CD69 and CD107a expression Elevate Elevate IFN-γ production	Tumor-bearing mice, cell lines	(75)
Recombinant mouse IL-15	Induce NK cell proliferation Increase cytotoxic activity of NK cells	Increase IFN-γ secretion	Tumor-bearing mice	(84)
Fractalkine (CX3CL1)	Increase NK cell infiltration Induce innate and adoptive immunity	Chemoattract NK cells	Human resections of gastric adenocarcinoma	(76)

### Immunomodulatory Drugs Tune the NK Cells Immunologic Function

Immunomodulatory drugs have made great progress in the treatment of cancer in recent years. Combined treatment of recombinant macrophage inflammatory protein-1 alpha and signaling bacterium acnes could recruit a large number of NK cells to both tumor sites and regional lymph nodes. Moreover, it induced a strong T-helper 1 immunity at an early time, which later led to improved survival of tumor-bearing mice (77). Alternatively, polysaccharide krestin (PSK), a mushroom extract, is a specific TLR2 agonist and could offer significant advantages in survival over chemotherapy alone for patients with curative resections of GCs (78). PSK was previously reported to mediate induction of the NKL cell proliferation and activation, and it was found to induce apoptosis in the AGS cell line as well (79). Of interest, a neutral polysaccharide fraction (SMPA) from salvia miltiorrhiza significantly promoted the production of anti-inflammatory cytokines (IL-2, IL-4, and IL-10) and augmented the killing activity of NK cells in GC rats (80). Lupeol, a triterpene found in various vegetables, increases the proliferation and killing effect of NK cells on GC cell lines BGC823, N87, and HGC27 by increasing the expression of perforin, IFN-γ, and CD107a via the activation of the PI3K/Akt and Wnt/β-catenin signaling pathways (81). Subsequently, Qu et al. unveiled a synthetic analog of double-stranded RNA intracellular poly(I:C), which not only triggered gastric adenocarcinoma cell apoptosis but also induced type I IFN production by gastric adenocarcinoma cells (82). In addition, the histone deacetylase inhibitor valproic acid could increase sensitivity to expanded NK cells by upregulating the expression of MICA/B (32).

### Immune Checkpoint Blockades Augment NK Cell-Mediated Lysis

Immune checkpoints are molecules that can provide either activating or inhibitory signals to the immune system. Stimulators CD28, OX40, CD58, CD40L, CD80, CD86, and CD137 can promote immune activation, whereas inhibitors programmed death-1 (PD-1), cytotoxic lymphocyte associated antigen-4 (CTLA-4), lymphocyte activation gene 3, T-cell immunoreceptor with Ig and ITIM domains, T-cell immunoglobulin, and mucin-domain containing-3 suppress immune activation (47, 83). Blockades targeting these checkpoints are being tested for the potential to treat cancer.

Two main checkpoints are PD-1 and CTLA-4, and therapeutic blockades of them have become a paradigm-shifting treatment in solid tumor oncology (85). Engagement of PD-1 with programmed death ligand 1 (PD-L1) expressed on cancer cells results in the suppression of T-cell proliferation and response, which eventually leads to tumor immune evasion (86). Accumulating data show that the expression of PD-L1 is upregulated in tumor cells from patients with GC, especially in mismatch repair-deficient and Epstein-Barr virus-positive GC, which suggests that the PD-1/PD-L1 pathway plays a critical role in the immune evasion of GCs (87–90). In addition, Liu et al. detected PD-1 expression on peripheral NK cells in patients with GC by flow cytometry. Compared with that in healthy controls, a significant increase in PD-1 expression on NK cells was observed in GC patients. More importantly, PD-1/PD-L1 blockades significantly augmented degranulation and IFN-γ secretion and suppressed apoptosis of NK cells by enhancing the activation of the PI3K/AKT signaling pathway in NK cells (91). Many clinical trials on the anti-PD-1 antibody pembrolizumab have been explored in the treatment of GC and were proven to be safe in the setting of design. In a phase 1b trial involving the use of pembrolizumab in patients with PD-L1-positive advanced GC, pembrolizumab had a manageable toxicity profile and promising antitumor activity, eliciting sustained antitumor responses in 22% of patients according to a central review (92). As a fully human anti-PD-L1 IgG1 antibody, avelumab obtained an acceptable safety profile but did not result in an improvement in OS or progression-free survival in patients with gastric or gastroesophageal junction cancer (GEJC) with single-agent avelumab in the third-line setting (93). Apart from anti-PD1/PD-L1 antibodies, ipilimumab and tremelimumab (which target CTLA-4) are also under clinical investigation in the treatment of GC. A recent study employing ipilimumab in unresectable locally advanced/metastatic GC/GEJC did not prove ipilimumab efficacy as monotherapy, whereas a comparable median OS of ~1 year and a favorable safety profile supported the investigation of ipilimumab in combination with other therapies for advanced GC (94). Tremelimumab as a second-line treatment for metastatic esophageal and gastric adenocarcinomas achieved only a 5% objective response rate in a phase II study. However, a small cohort of patients (4 of 18) achieved disease control, as assessed by stable computed tomography scan (95). Nevertheless, effects of anti-PD-1/PD-L1 antibodies on NK cells in patients with GC need further evaluation.

### Antibodies Increase NK Cell Cytotoxicity to GC via ADCC

Immunotherapy of tumors with specific antibodies has achieved great success in the past 20 years. Herceptin is a humanized monoclonal antibody (mAb) that specifically targets human epidermal growth factor receptor-2 (HER2)/neu and exhibits growth inhibitory activity against HER2/neu-overexpressing tumors. Research has suggested that HER2/neu-expressing GC cells could be killed by Herceptin-mediated ADCC and depend on the degree of HER2/neu expression on the GC cells. However, Herceptin-mediated ADCC was significantly impaired because of its NK cell dysfunction in patients with advanced disease. Interestingly, IL-2 *ex vivo* treatment of NK cells could restore CD16zeta expression, contributing to restoration of Herceptin-mediated ADCC (31). Furthermore, Mimura et al. discovered that the presence of Herceptin markedly increased the cytotoxicity of expanded NK cells against the HER2-positive GC cell lines MKN7 and NCI-N87. Meanwhile, lapatinib, which targets both HER2 and epidermal growth factor receptor (EGFR), could upregulate HER2 cell surface expression on both MKN7 and NCI-N87, resulting in an increase in Herceptin-mediated ADCC by expanded NK cells (32). Another antibody, cetuximab, is a chimeric mAb to EGFR. Hara et al. demonstrated that cetuximab showed moderate antitumor activity to MKN-28 cells by slightly inhibiting ligand-induced phosphorylation of protein kinase B and extracellular signal-regulated kinase, but cetuximab in combination with IL-2 significantly inhibited subcutaneous and intraperitoneal tumor growth of MKN-28 cells in nude mice by NK cell-mediated ADCC rather than the blockade of the intracellular signaling pathway (96). Besides, Hasegawa et al. produced an afucosylated humanized anti-EPHA2 mAb DS-8895a, which could recognize and bind to EPHA2 that was anchored to cell membranes. DS-8895a markedly enhanced NK cell-mediated ADCC *in vitro* and also inhibited tumor growth in EPHA2-positive human GC SNU-16 xenograft mouse models (97). Moreover, an FGFR2b-specific humanized monoclonal antibody, FPA144, has been investigated to treat patients with GC overexpression of the FGFR2b as a single agent in clinical trials (NCT02318329). FPA144 not only blocks ligand binding and induces FGFR2b internalization but also enhances ADCC. Notably, FPA144 increased PD-L1-expressing cells in the tumor microenvironment, and the combination of FPA144 and RPM1-14, a PD-1 blockade, inhibited tumor growth by 49% (*p* < 0.001) (98). All the above studies suggest that using mAb to increase NK cell activity would be a promising alternative approach for a subset of GC patients, even though most are not yet available for routine use in GC.

### Vaccines Stimulate Antitumor Immune Responses Against GC

Vaccines have been a useful tool to stimulate both adaptive and innate antitumor immune responses to improve cancer immunotherapy. As DCs are professional antigen-presenting cells that can capture and process tumor-associated antigens, DC-based vaccines evolved as promising vaccination protocols in cancer therapy. Schmitz et al. revealed that M-DC8^+^ DCs could stimulate proliferation, IFN-γ secretion, and tumor-directed cytotoxicity of NK cells depending on cell-to-cell contact (99). Liu et al. pulsed DCs with total RNA from MFC GC cells as vaccine and discovered that it stimulated and upregulated NK cells and tumor-specific CTL activity in mice with GC xenograft, highlighting that the use of DC-based tumor vaccine could provide a glimmer of hope for patients with GC (100). HSP-gp96 is a heat shock protein glycoprotein named according to its molecular weight of 96 kDa. Lu et al. infected human GC cell lines KATOIII, MKN-28, and SGC-7901 with adenovirus gp96 at a multiplicity of infection of 100 and purified gp96-GC antigen peptide complexes. Compared with GC-derived peptide, gp96-GC antigen peptide complexes markedly improved NK cell activity at different concentrations (101).

### Gene Therapy Improves the Immunogenicity or Susceptibility of Gastric Tumor Cells to NK Cells

Gene therapy offers a new measure for GC treatment. Intercellular adhesion molecule (ICAM)-2 is a second ligand of leukocyte function-associated antigen-1 (CD11a/CD18). The interaction between CD11a/CD18 and ICAM-2 can mediate many leukocyte functions, including Ig production and the cytotoxicity of NK cells. Tanaka et al. gave mice with peritoneal dissemination of scirrhous gastric carcinoma an injection of an adenovirus vector, AdICAM-2, that encoded the full-length human ICAM-2 gene. The tumor-bearing mice survived for a significantly longer time, and many NK cells filtrated the peritoneal metastatic lesions, indicating ICAM-2 transfection might be an effective form of gene therapy for peritoneal metastasis of GC (102). Moreover, Li et al. transfected the MGC GC cell line with small interfering RNA (siRNA) that could silence the expression of heavy chain genes of all immunoglobulin isotypes consistently. The siRNA could knock down cancerous Ig, which inhibited ADCC by competitively binding to the Fc receptor on NK cells. As a result, it enhanced ADCC induced by an EGFR antibody in a dose-dependent manner and inhibited the growth of MGC GC cells (103).

## Conclusion

As essential effectors in host immunity, NK cells can mediate the death of GC cells by ADCC, releasing perforin and granzymes, secreting IFN-γ and TNF-α, or eliciting apoptosis via formation of complex FAS/FASL and TRAIL/TRAILR. NK cells' activity is correlated to clinical stage, lymphatic and vascular invasion, lymph node metastases, and prognosis in GC patients. Gastric tumors could escape NK cell surveillance via downregulating ligands of activating receptors, secreting suppressive cytokines, and attracting suppressive cells. With the progression of GC, both the number and activity of NK cells are decreased. Thus, reversing NK cell dysfunction may be an effective treatment for GC.

As mentioned earlier, NK cell adoptive therapy is a safe and well-tolerated procedure, but it showed limited value because of the limited number of available NK cells. Expanded NK cells for adoptive treatment has become a promising measure to solve the problem. However, there is little research investigating whether expanded NK cells for adoptive treatment is effective for GC patients. A phase I clinical trial determined that autologous expanded NK cell therapy was safe and well-tolerated in patients with advanced digestive cancer, including GC. Although NK cell transfer as a monotherapy did not result in a clinical response in patients, transferred NK cells persisted in the peripheral circulation of patients and exerted cytotoxicity *in vitro*, providing the potential of efficacious combination treatment with other reagents (69). Combination therapy of adoptive NK cell therapy and IgG1 monoclonal antibodies shows good tolerability and preliminary antitumor activity in patients with unresectable advanced gastric or colorectal cancer along with induced Th1-type immune response and reduced peripheral Tregs (104). Simultaneously, some cytokines, immunomodulatory drugs, immune checkpoint blockades, antibodies, vaccines, and immunogene therapies can enhance NK cell function through different mechanisms and have made some achievements in inhibiting the growth of GC in some studies. But further research is still needed to optimize NK cell-based therapy. A combination of different treatment strategies may make promising outcomes. In recent years, chimeric antigen receptor (CAR)-modified NK cells have appeared as a revolutionary immunotherapy option for the treatment of many malignancies (105, 106). CARs consist of an extracellular single-chain variable fragment capable of recognizing a cancer antigen and intracellular activation motifs that activate NK cytotoxicity upon antigen recognition. With the addition of a CAR, NK cells might add a new method of redirecting target cells to increase the number of NK cells in tumor regions. It is specifically useful in patients with downregulated activating receptors. CAR-NK may provide a new research direction for GC. CAR-NK can be obtained from umbilical cord blood gene-modified human hematopoietic stem cells using co-culture with a feeder stroma of murine OP9-DL1 cells in the presence of human recombinant cytokines or using insulin-like growth factor 1 alone (67). Recognizing the low transfection efficiency of blood NK cells, investigators are trying to generate a clonal NK-cell line. At present, only the NK-92 cell line displays a consistent and high cytotoxicity to cancer targets. NK-92 cells can be easily engineered by non-viral transfection methods to express CARs that can retarget them toward malignant cells. In addition, the preparation and administration of NK-92 cost significantly less compared with autologous or allogeneic NK cells and, particularly, compared with CAR-T cells (107). CAR-NK cells specific for CD19, CD20, EGFR, and HER2 have made promising progress in killing of target cells. Of note, CAR designs with 4-1BB co-stimulation led to a higher cytolytic capacity and cytokine production (108). Given this, HER2+ GC may benefit from treatment of CAR-NK cells. Nevertheless, our review suggests that NK cell-based therapy is expected to offer a promising prospect to GC patients and deserves more study.

## Author Contributions

YW conceived this study and YD wrote the manuscript. All authors revised the manuscript.

### Conflict of Interest Statement

The authors declare that the research was conducted in the absence of any commercial or financial relationships that could be construed as a potential conflict of interest.

## Abbreviations

GC: gastric cancer
NK cells: natural killer cells
TGF: transforming growth factor
IL: interleukin
ADCC: antibody-dependent cellular cytotoxicity
KIRs: killer-cell immunoglobulin-like receptors
HLA: human leukocyte antigen
TNF: tumor necrosis factor
TRAIL: TNF-related apoptosis inducing ligand family
FASL: Fas-ligand
IFN: interferon
MDSCs, macrophages: myeloid-derived suppressor cells
MHC: major histocompatibility complex
MICA: MHC class I chain-related A
GVHD: graft-vs-host disease
EBV-LCL: Epstein-Barr virus-transformed lymphoblastoid cell lines
GMP: good manufacturing practices
PBMC: peripheral blood mononuclear cell
PSK: polysaccharide krestin
PD-1: programmed death 1
PD-L1: programmed death ligand 1
CTLA-4: cytotoxic lymphocyte-associated antigen 4
GEJC: gastroesophageal junction cancer
mAb: monoclonal antibody
HER2: human epidermal growth factor receptor-2
EGFR: epidermal growth factor receptor
DCs: dendritic cells
ICAM: intercellular adhesion molecule
siRNA: small interfering RNA
CAR: chimeric antigen receptor
OS: overall survival
TAM: tumor-associated macrophage.
